# Characterization of the effect of LPS on dendritic cell subset discrimination in spleen

**DOI:** 10.1111/jcmm.12332

**Published:** 2014-06-10

**Authors:** Kristin L Griffiths, Jonathan KH Tan, Helen C O'Neill

**Affiliations:** Research School of Biology, The Australian National UniversityCanberra, ACT, Australia

**Keywords:** dendritic cell, lipopolysaccharide, spleen

## Abstract

The Gram-negative bacterial endotoxin lipopolysaccharide (LPS) is a potent inflammatory mediator and a leading cause of bacterial sepsis. While LPS is known to activate antigen-presenting cells, here we find that LPS down-regulates expression of CD11c and CD11b on splenic dendritic cell subsets, thus confounding the ability to identify these subsets following treatment. This has implications with regard to tracking the response to LPS in terms of the cell subsets involved, and should be considered whenever such studies are undertaken.

## Introduction

Dendritic cells (DCs) are often considered the sentinels of the immune system, acting as antigen-presenting cells (APC) with the primary role of taking up antigen and presenting it to T cells to initiate an adaptive immune response. It is now accepted that DCs can be divided into several different subsets based on marker expression, function and tissue/organ of residence (reviewed by Merad *et al*. [1]). Two of the key markers used to identify dendritic and myeloid cells from other leucocytes are CD11b and CD11c. As β_2_ integrins, both exist as a heterodimer with CD18 on the cell surface [2], and both are thought to have roles in adhesion to endothelial cells and in complement recognition. CD11b, in particular, has been implicated in leucocyte rolling and crawling, mechanisms employed to capture and slow leucocytes flowing through blood vessels to facilitate extravasation [3]. In relation to DC identification, CD11c and CD11b are used as two of the primary markers for delineation of splenic DC subsets [1].

The Gram-negative bacterial endotoxin lipopolysaccharide (LPS) is known to be a potent inflammatory mediator. Here, we use LPS as a model toxin to induce inflammation *in vivo* to investigate the effects of the toxin on marker expression and prevalence of APC subsets in the spleen. Ability to identify APC subsets throughout the lymphoid system is important for the dissection of the immune response to infection. This paper identifies shortcomings of performing analyses of APC subset representation under inflammatory conditions.

## Materials and methods

### Animals

Specific-pathogen free 6-week-old female C57BL/6J mice were obtained from the John Curtin School of Medical Research (Australian National University, Canberra, Australia). Mice were housed and handled according to protocols approved by the Animal Experimentation Ethics Committee at the Australian National University and killed by cervical dislocation.

### LPS treatment of mice

Mice were treated through *i.v*. injection of LPS in HBSS at a dose of 0.1 μg/g bodyweight. Injections were performed into the tail vein of mice placed in a restraint by using a 1-ml syringe equipped with a 26G needle.

### Preparation of lymphoid cells

For preparation of single-cell suspensions, dissected spleen or mesenteric lymph nodes were pressed through a fine mesh sieve. Cells were resuspended in 5 ml DMEM supplemented with 10% Foetal Calf Serum (JRH Biosciences, Lenexa, KS, USA), 10 mM Hepes (JRH Biosciences), 2 mM L-glutamine (JRH Biosciences), 100 μg penicillin/streptomycin and 5 × 10^−5^ M 2-mercaptoethanol (BDH Ltd., Poole, UK; sDMEM). Cells were then sedimented, and splenocytes were treated with RBC lysis buffer (140 mM NH_4_Cl, 17 mM Tris base) and washed.

### MACS separation

Enrichment of DC populations was performed by depletion of T and B cells from a cell suspension of dissociated spleen. Cells were incubated with 0.25 μg/ml biotinylated anti-CD19 (B cells: eBioscience; San Diego, CA, USA) and 0.2 μg/ml biotinylated anti-Thy1.2 (T cells: eBioscience) antibody per 10^8^ cells in 1 ml. Antibody was diluted in MACS labelling buffer (PBS/2 mM EDTA/5% BSA) and absorbed to cells for 10 min. on ice Cells were washed twice with labelling buffer before addition of anti-biotin magnetic microbeads (Miltenyi Biotec, Auburn, CA, USA). Cells were absorbed with 13 μl beads/10^8^ cells in 1 ml for 25 min. on ice. After washing with MACS labelling buffer, cells were resuspended in 500 μl of buffer. The cell suspension was then run through a pre-washed LS column (Miltenyi Biotec) positioned in the strong magnetic field of a SuperMACS II Separator (Miltenyi Biotec), which retains cells with bound magnetic microbeads. Splenocytes depleted of T and B cells were collected as flow-through cells, along with cells collected after three column washes with 3 ml labelling buffer. These cells were resuspended in sDMEM for staining.

### Antibody staining of cells

Cells were sedimented into the wells of a flexible 96-well polystyrene microtitre plate (Corning, New York, USA). For FcR blocking, cells were resuspended in 25 μl anti-CD16/32 followed by incubation on ice for 15 min. After washing, cells were resuspended in a primary antibody cocktail (APC-conjugated CD11c (N418), PE-Cy7-conjugated CD11b (M1/70), PE-conjugated CD8α (53-6.7), biotin- or FITC-conjugated MHC-II (AF6-120.1) all from eBioscience) and incubated on ice for 25 min. before washing. Where necessary, secondary labelled conjugate (streptavidin-APC-Cy7 from eBioscience) was added and cells were incubated for 25 min. on ice, washed and transferred to a cluster tube (Corning) for analysis performed with a flow cytometer (Becton Dickinson LSRII, San Jose, CA, USA). Prior to analysis, propidium iodide (PI: 1000 μg/ml) was added for dead cell discrimination.

### Flow cytometry

Flow cytometry was performed with an LSRII flow cytometer (Becton Dickinson). Background binding of each antibody was determined by using isotype control antibodies corresponding to each fluorescence-conjugated antibody used, and gates were set by using this information as well as internal positive and negative cell subsets. Live cells were detected by absence of staining with PI. Analysis was performed with BD FACSDiva Software (Becton Dickinson) and Flow Jo software (Tristar, Phoenix, AZ, USA).

### Endocytic capacity of cells

Ovalbumin (OVA)-FITC (100 μg/g bodyweight) was injected *i.v*. into mice in HBSS. Mice were left for 24 hrs before killing and spleen removal. Spleens were dissociated and stained with antibodies as above. Endocytosis was measured as% FITC^+^ cells.

## Results

### LPS induces down-regulation of CD11c and CD11b expression on DC populations in spleen

**Fig. 1:**
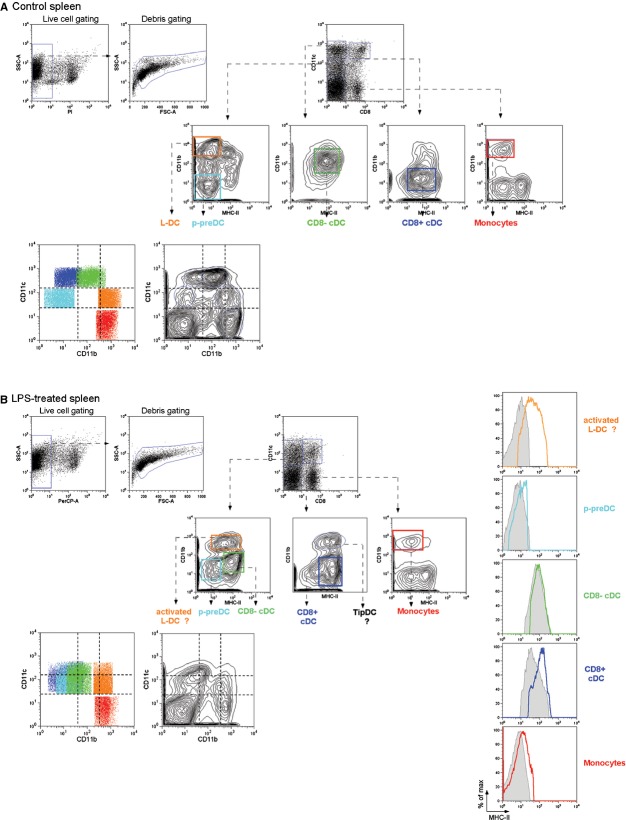
Gating protocol used to assess the effect of LPS on marker expression on myeloid and dendritic cell subsets in spleen. (**A**) Spleens from 6-week-old C57BL/6J mice were depleted of T and B cells by using magnetic bead separation and stained by using fluorochrome-conjugated antibodies for analysis by flow cytometry. After dead cell exclusion based on propidium iodide (PI) staining, dendritic and myeloid cell subsets were identified by gating on CD11c *versus* CD8 and then CD11b *versus* MHC-II plots as described in Tan *et al*. [4]. Each identified population was then overlaid on a CD11b *versus* CD11c plot. (**B**) Six-week-old C57BL/6J mice were injected *i.v*. with LPS (0.1 μg/g bodyweight) 24 hrs prior to killing. Splenocytes were prepared and gating performed as described above. Changes in MHC-II expression on subsets is revealed by overlaid histograms: treated (coloured line); untreated (grey filled).

In this paper, we take steady-state splenic dendritic and myeloid cell subsets to include CD8α^+^ and CD8α^−^ conventional DCs (cDCs), plasmacytoid-pre-DCs (p-preDCs), monocytes and L-DCs (described by Tan *et al*. in 2011 [4]). Figure 1A shows clear identification of splenic dendritic and myeloid subsets on a CD11c *versus* CD11b plot in untreated C57BL/6J mice, confirming that in the steady-state, each splenic dendritic/myeloid subset has unique CD11c and CD11b expression levels.

We next investigated the effect of endotoxin treatment on surface expression of CD11c, CD11b, CD8α and MHC-II on splenic dendritic and myeloid cell subsets. C57BL/6J mice were administered LPS *i.v*. and subsets analysed as above. As shown in Figure 1B, the greatest effect of LPS treatment on cDC subsets was to reduce levels of both CD11c and CD11b expression. This resulted in the cDC subsets being indistinguishable on the basis of CD11c and CD11b expression. L-DCs and monocytes have low or no CD11c expression, respectively, and this remains the case following LPS treatment except for a slight increase in CD11c expression on a subset of L-DC. Similarly, these subsets maintain high, but slightly lower, levels of CD11b expression. LPS treatment up-regulated MHC-II on the cell surface of all subsets except CD8^−^ cDCs in terms of expression of markers investigated here (Fig 1B and Table 1).

**Table 1:** MHC-II expression increases following LPS treatment

DC subset	MHC-II (MFI: mean ± SD)†	

	Control (*n* = 7)	LPS (*n* = 5)
CD8α+ cDCs	59.7 ± 16.0	231.0 ± 69.6***
CD8α- cDCs	80.0 ± 21.8	122.1 ± 45.4
p-preDCs	3.7 ± 0.3	9.73 ± 1.8***
Monocytes	2.8 ± 0.4	6.7 ± 2.2**
L-DCs	3.1 ± 2.4	40.1 ± 14.8***

† MFI: mean fluorescence intensity.

** *P* < 0.01,

*** *P* < 0.001.

### LPS decreases relative percentage of p-preDCs in spleen

**Fig. 2:**
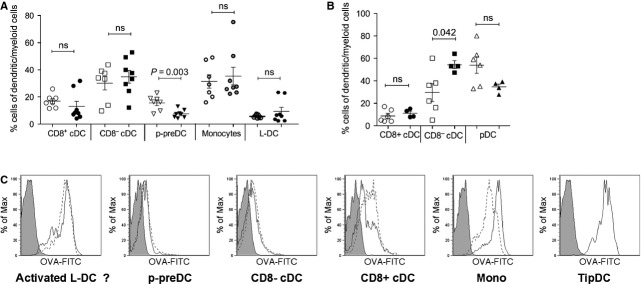
The effect of LPS on prevalence and endocytic capacity of dendritic and myeloid subsets. (**A**) Splenocytes from LPS-treated (closed symbols) and untreated (open symbols) animals were prepared, stained and analysed by flow cytometry as described for Figure 1. Percentage representation of each dendritic and myeloid subset relative to total dendritic and myeloid cells was calculated and depicted graphically. (**B**) Mesenteric lymph nodes from LPS-treated and untreated animals were prepared, stained and gated as described for spleens, with the exception that no T- and B-cell depletion was performed. Relative percentage of each dendritic and myeloid subset was calculated and graphed. (**C**) C57BL/6J mice were given OVA-FITC (100 μg/g bodyweight) along with LPS (0.1 μg/g bodyweight) at 24 hrs prior to killing. Splenocytes were prepared as described in Figure 1, and following identification of each dendritic and myeloid subset, uptake of OVA-FITC was measured. Dotted lines represent OVA-FITC uptake by cells from untreated mice; solid lines represent OVA-FITC uptake by cells from LPS-treated mice; solid histogram represents background FITC level for each subset from animals receiving OVA alone (no FITC).

To determine the effect of endotoxin treatment on the relative prevalence of splenic dendritic and myeloid cell subsets, representation of each subset as a percentage of the total dendritic and myeloid population in spleen was calculated (Fig. 2A). Interestingly, none of the mature subsets showed any change in prevalence following LPS treatment. Relative percentages of p-preDCs, on the other hand, were reduced under conditions of simulated inflammation (Fig. 2A). To assess possible p-preDC migration to lymph nodes as mature pDCs, percentages of CD8α^+^ cDCs, CD8α^−^ cDCs and pDCs in mesenteric lymph nodes were determined. Results showed an increased percentages of CD8α^−^ cDCs and a small, but non-significant, decrease in pDCs (Fig. 2B).

### The effect of LPS on *in vivo* endocytic capacity of cells

We next investigated the effect of LPS treatment on the endocytic capacity of dendritic and myeloid cells in the spleen. Mice were administered LPS *i.v*. with or without FITC-conjugated ovalbumin (OVA-FITC). Spleens were taken 24 hrs later and the amount of OVA-FITC taken up by each cell subset examined by flow cytometry. Results (Fig. 2C) showed that, with the exception of monocytes and CD8α^+^ cDCs, LPS had no effect on the *in vivo* endocytic capacity of cells. Monocytes showed some increase in uptake of OVA-FITC, while CD8α^+^ cDCs had slightly reduced endocytic capacity following LPS treatment. The increase in phagocytic capacity of myeloid cells could reflect a form of maturation towards a macrophage induced by LPS, whose primary function is phagocytosis for destruction of an invading pathogen. Indeed, functionally, LPS has been shown to induce phagocytosis in macrophages [5,6], an observation supported by data presented here. It is likely, however, that CD8α^+^ cDCs, on the other hand, respond to maturation by reducing levels of phagocytosis so as to divert energy into antigen presentation following encounter with the pathogen.

## Discussion

We show here that LPS has the ability to down-regulate surface expression of CD11c and CD11b on cDC subsets in the spleen. CD11c/CD18 and CD11b/CD18 are integrins expressed on the cell surface involved in cell adhesion and migration. They also bind components of the complement system and are therefore involved in pathogen sensing [7]. Furthermore, there are reports to suggest that CD11b/CD18 forms part of a CD14-mediated cell surface cluster in response to LPS [8,9], and that CD11b/CD18 and CD11c/CD18 can induce signalling following LPS treatment independently of CD14 [10,11]. Thus, down-regulation of CD11c and CD11b is probably a direct effect of LPS acting through the CD11b/CD11c binding, as well as a reflection of an LPS-induced shift in cellular function.

Our observation of down-regulation of CD11c under inflammatory conditions is consistent with a recent report that CD11c on BM-derived DCs cultured *in vitro* is down-regulated following TLR activation, as occurs with LPS treatment [12]. This could reflect preparation of the cells for migration from the spleen to the periphery following recognition of a danger signal. Investigation of cellular migration was not in the scope of this paper; however, LPS-induced migration of cDC subsets from the spleen may be accompanied by replacement with mature BM precursors, consistent with no observed perturbation of cell numbers in spleen after LPS treatment. The same paper reported an increase in MHC-II expression following Toll-like receptor activation. Our results confirm this observation following an *in vivo* challenge with LPS, an outcome that would improve the antigen-presenting capability of cells following activation.

Relative proportions of mature dendritic and myeloid cells in spleen were not affected by LPS treatment; however, the proportional representation of p-preDCs was lower in LPS-treated mice. As p-preDCs represent an immature, inactivated form of pDCs, a drop in their numbers could reflect migration from the spleen to other organs. To test whether pDCs had migrated to other sites, we investigated mesenteric lymph nodes and saw no increase in numbers of pDCs. However, we did not progress to determine their presence in other lymph nodes or blood. The assumption is that changes in their migratory capacity may have led to dissemination of cells as a result of LPS treatment.

Results presented here have important implications for the *in vivo* study of the innate immune response to infection, particularly if individual subsets responsible for a certain response are of interest. As we have shown, in the steady-state, splenic APC subsets are readily identifiable based on CD11b and CD11c expression, and can further be defined by CD8 and MHC-II expression. Following exposure to LPS, however, expression of these key markers changes, probably as a representation of the altered function of the cells under inflammatory conditions. These changes result in difficulties in identifying individual APC subsets, a factor that should be considered during analysis of an *in vivo* response to infection.
